# Nutritional status, health risk behaviors, and eating habits are correlated with physical activity and exercise of brazilian older hypertensive adults: a cross-sectional study

**DOI:** 10.1186/s12889-022-14873-4

**Published:** 2022-12-19

**Authors:** Leonardo Santos Lopes da Silva, Daniel de Freitas Batalhão, Anderson dos Santos Carvalho, Lucimere Bohn, Nilo César Ramos, Pedro Pugliesi Abdalla

**Affiliations:** 1 School of Physical Education and Sport of Ribeirão Preto, University of São Paulo, Bandeirantes Avenue nº 3900, University Campus - Monte Alegre, Ribeirão Preto-SP, 14030-680 Brazil; 2 Study and Research Group in Anthropometry, Training, and Sport (GEPEATE), School of Physical Education and Sport of Ribeirão Preto, University of São Paulo, Ribeirão Preto, Brazil; 3 Physical Education Department, Paulista University, São José Do Rio Preto, Brazil; 4 Faculty of Sports (FADEUP), University of Porto, Porto, Portugal; 5 Research Center for Physical Activity, Health and Leisure (CIAFEL), University of Porto, Porto, Portugal; 6 Laboratory for Integrative and Translational Research in Population Health (ITR), Porto, Portugal; 7 Faculdade de Psicologia, Educação e Desporto, University Lusófona of Porto, Porto, Portugal; 8 Coastal Carolina University, Conway, SC USA

**Keywords:** Arterial hypertension, Aging, Sedentary behavior, Heart disease risk factors

## Abstract

**Background:**

Nutritional status, health risk behaviors, eating habits, and other comorbidities (such as diabetes) may be associated with recommended amounts of physical activity (PA) and exercise (EX) in healthy older adults. However, these associations are still unclear for older hypertensive adults, who require greater care from health professionals. The purpose of this study was to associate the nutritional status, health risk behaviors, eating habits, and the presence of diabetes with recommended amounts of physical activity and exercise practice of older hypertensive adults.

**Methods:**

Ten thousand seven hundred eighty-nine older hypertensive adults (70.9 ± 7.4 years) from the VIGITEL telephone survey were classified according to PA levels (insufficiently active/sufficiently active) and EX practice (non-practitioners/practitioners). Binary logistic regression was used to observe the odds ratio (OR) between independent variables (nutritional status [body mass index], sociodemographic characteristics [age/sex/years of study], risk behaviors [screen time/alcohol/tobacco consumption], eating habits [minimally/ultra-processed foods consumption score], and the presence of diabetes) with recommended amounts of PA/EX (dependent variable).

**Results:**

Highest nutritional status (OR_PA_ = 0.975 [95%-CI: 0.965 – 0.985]; OR_EX_ = 0.981[95%-CI: 0.972 – 0.991]), age (OR_PA_ = 0.955 [95%-CI: 0.949 – 0.961]; OR_EX_ = 0.980[95%-CI: 0.975 – 0.986]), screen time (OR_PA_ = 0.909[95%-CI: 0.835 – 0.990]), alcohol consumption (OR_PA_ = 0.683[95%-CI: 0.621 – 0.758]; OR_EX_ = 0.702[95%-CI: 0.637 – 0.779]), tobacco (OR_PA_ = 0.601 [95%-CI: 0.492 – 0.736]; OR_EX_ = 0.464[95%-CI: 0.384 – 0.562]) ultra-processed foods consumption score (OR_PA_ = 0.896[95%-CI: 0.871 – 0.921]; OR_EX_ = 0.886[95%-CI: 0.863 – 0.909]) and having diabetes (OR_PA_ = 0.780[95%-CI: 0.708 – 0.859]; OR_EX_ = 0.831[95%-CI: 0.759 – 0.909]) reduced the odds of being sufficiently active/practicing exercise (*p* < 0.05). Male sex (OR_PA_ = 1.633[95%-CI: 1.491 – 1.789]; OR_EX_ = 1.247[95%-CI: 1.140 – 1.363]), years of study (OR_PA_ = 1.026[95%-CI: 1.018 – 1.035]; OR_EX_ = 1.050[95%-CI: 1.041 – 1.058]), and minimally processed foods consumption score increased the odds of being sufficiently active/practicing exercise (OR_PA_ = 1.132[95%-CI: 1.109 – 1.155]; OR_EX_ = 1.167[95%-CI: 1.145 – 1.191], respectively; *p* < 0.05).

**Conclusion:**

Nutritional status, health risk behaviors, eating habits, and the presence of diabetes were associated with the odds of older hypertensive adults complying with PA and EX recommendations. The results may help health professionals understand how these factors are associated with the changes of older hypertensive adults participating in physical activity and exercise.

**Supplementary Information:**

The online version contains supplementary material available at 10.1186/s12889-022-14873-4.

## Background

Aging is characterized by the progressive loss of adaptive capacity, which leads to a subsequent reduction in functionality [1]. Over the last few decades, geriatric populations have been occupying an increasingly high percentage of the demographic makeup of many countries [2, 3]. At the same time, an increase in noncommunicable diseases has been observed, responsible for the mortality and morbidity of older adults [4]. Among these diseases, cardiovascular diseases are the most prevalent in the world [5], and arterial hypertension is the main risk factor [4].

The first line of treatment for arterial hypertension, regardless of severity, focuses on the adoption of healthy lifestyles [1, 6]. Physical activity and exercise have been recommended as effective non-pharmacological methods of arterial hypertension control, contributing to the management of cardiovascular risk and quality of life of older adults [2, 3, 7–13]. Physical activity is characterized by any body movement produced by muscle contraction, resulting in higher energetic expenditure compared to basal rest levels [13]. Exercise is a subtype of physical activity that is characterized by the execution of planned movements, structured and performed to respond to a specific objective [13]. The benefits provided by regular physical activity and exercise on cardiovascular health are widely recognized [9] and support the international physical activity recommendations of the World Health Organization [14]. Supplementary Chart 1 reports detailed physical activity and exercise recommendations for cardiovascular benefits of older hypertensive adults. Briefly, recent evidence suggests a decrease in systolic and diastolic blood pressure and positive changes in metabolic profile with aerobic exercise (with an intensity of 70–80% of maximum oxygen consumption) [10]. For strength exercises, a decrease in systolic blood pressure (intensity of load: 9–15 maximum repetitions) and positive changes in oxidative stress and forearm vasodilatation (intensity of load: 6–10 maximum repetitions) [11, 12] were observed. For combined exercises (both aerobic and strength), positive changes in metabolic profile (intensity: 55–85% of maximal heart rate) and a decrease in arterial stiffness (intensity: 60–75% of maximal heart rate) are highlighted [7, 10].

Although many older adults exercise regularly, a significant amount does not seem to meet the 150 min of moderate to vigorous physical activity recommended by the World Health Organization [15, 16]. According to data from the Behavioral Risk Factor Surveillance System 2013 (sample of 139,723 older adults from a high-income country), approximately 45% of older adults meet the physical activity guidelines, and 36% are not enrolled in exercise programs [16]. For middle/low-income countries, the effort and compliance of older adults who meet the physical activity recommendations and are enrolled in exercise programs appear to be relatively low [17, 18]. Approximately, only 31% meet the physical activity guidelines and 76% are not enrolled in exercise programs [17, 18].

Some behavior factors can increase the odds of older hypertensive adults adhering to exercise programs and being sufficiently active [19–22]. These factors include nutritional status, screen time, alcohol and tobacco use, and food (minimally/ultra-processed) consumption [19, 20]. Strong evidence demonstrates that these factors can dictate the adherence of older hypertensive adults to exercise programs and physical activity recommendations [21, 22]. However, the evidence is still limited and restricted to samples of young adults and older European healthy adults (from Portugal and New South Wales, respectively) [21, 22]. Older Brazilian hypertensive adults may be quite different from older Europeans due to biological, cultural, geographical, and sociodemographic reasons.

Thus, the purpose of this study was to verify the association of nutritional status, health risk behaviors, eating habits, and the presence of diabetes on recommended amounts of physical activity and exercise of older hypertensive adults. We hypothesized that nutritional status, health risk behaviors eating habits, and the presence of diabetes were associated with the odds of older hypertensive adults engaging in sufficient levels of physical activity and exercise.

## Methods

### Study design and sample

The study design was observational and cross-sectional. The data originated from the epidemiological project called Estudo de Vigilância de Fatores de Risco e Proteção para Doenças Crônicas por Inquérito Telefônico [Surveillance of Risk and Protection Factors for Chronic Diseases by Telephone Survey] (VIGITEL). VIGITEL is conducted every year between January and December by the Ministry of Health in partnership with the Group of Studies, Research and Practices in Food and Health Environment of the Universidade Federal de Minas Gerais (GEPPAAS/UFMG) and the Nucleus for Epidemiological Research in Nutrition and Public Health of Universidade de São Paulo (NUPENS/USP). For this study, data from 2019 was used since this was the last year before the COVID-19 pandemic.

VIGITEL data collection is conducted through telephone survey systems with voluntary subjects between 18 and 106 years old, residents in the 26 Brazilian state capitals, and the Federal District. All subjects who have at least one telephone landline (52,443 participants) are eligible to participate in the survey. In short, participants (living in the 26 state capitals and Federal District) were called (recruited) randomly and those who completed the survey were selected as participants.

### Participants selection in the present study

The inclusion criteria for participants in the study were: a) ages between 60 and 90 years old; b) arterial hypertension diagnosis; c) recorded body mass (kg) and height (m); and d) available information on physical activity and exercise, education level, screen time, alcohol and tobacco consumption, minimally and ultra-processed foods consumption, and the presence (or not) of diabetes. We only selected participants between 60 and 90 years old because nonagenarians and the oldest are in a situation of extreme longevity [23, 24]. Some regions in the world showed that extreme longevity is not universal [25]. In this sense, nonagenarians and the oldest should be studied separately, especially for the present study that involves behavioral factors [26].

In this sense, from a random sample (52,443), all participants that fit the inclusion criteria mentioned above were selected for our study.

### Variables in the study

#### Sociodemographic characteristics

Sociodemographic variables considered for the study were chronologic age, sex, and education level (years of study), self-reported at the time of the interview. Years of education were included with the question “Up to what grade did you study?”, and the responses were reported in years (never studied; 1–12 or over [refers to postgraduate]) (p. 117 of [20]).

#### Anthropometry

Body mass (kg) and height (m) were self-reported during the phone interview (p. 117 of [20]). Body mass index (BMI) was subsequently calculated as kg/m^2^. Nutritional status was classified according to Lipschitz (1994) into three categories (underweight = BMI < 22 kg/m^2^; eutrophic = BMI 22 to 27 kg/m^2^; overweight = BMI > 27 kg/m^2^) [20].

#### Health risk behavior

Screen time (watching television and use of cell phone, tablet, or computer) was self-reported: a) “On average, how many hours a day do you usually spend watching TV?”; and b) “On average, how many hours of your free time (excluding work) do you spend on the computer, tablet or cell phone per day?” (p. 125 of [20]). Older adults were classified as spending ≥ 3 h/day on screen (‘yes’ and ‘no) [27]. Smoking (“Currently, do you smoke?”) (p. 126 of [20]) and alcohol consumption (“Do you usually consume alcoholic beverages?”) (p. 122 of [20]) were obtained as binary variables ('yes' or 'no').

#### Eating habits

Based on one question of the VIGITEL questionnaire (24-h recall method) “Now I'm going to list some foods and I would like you to tell me if you ate any of them yesterday (from when you woke up until when you went to sleep)” (p. 120–121 of [20]), food consumption score was recorded according to the daily presence of different foods. The 24-h recall method is a valid and reliable tool for assessing habitual intake of foods (energy and macronutrients at the group level) [28].

Foods were further stratified according to NOVA classification into groups of similar characteristics as minimally (obtained from plants or animals, without big changes in natural state until consumption) and ultra-processed (formulations of ingredients that result from a series of industrial processes, creating highly profitable, convenient, and hyper-palatable products) foods [29]. The foods were stratified into representative subgroups of similar features. For minimally processed foods, twelve subgroups were considered: 1) lettuce, kale, broccoli, watercress, or spinach; 2) pumpkin, carrot, sweet potato, or okra/caruru; 3) papaya, mango, yellow melon, or souari nut; 4) tomato, cucumber, zucchini, eggplant, chayote or beetroot; 5) orange, banana, apple or pineapple; 6) rice, pasta, polenta, couscous or corn; 7) beans, peas, lentils or chickpeas; 8) potato, cassava or yam; 9) beef, pork, chicken or fish; 10) fried, boiled or scrambled egg; 11) milk; 12) peanuts, cashew nuts or Brazil nuts. For example, if a person consumes lettuce and kale (both in 1° subgroup), a score of 1 is obtained. But, if consuming lettuce, kale (both in 1° subgroup), and pumpkin (2° subgroup), at score of 2 is obtained. For ultra-processed foods, thirteen subgroups were considered: 1) soda; 2) fruit juice in a box or can; 3) powdered refreshment; 4) chocolate drink; 5) flavored yogurt; 6) packet snacks or crackers/cookies; 7) biscuit/sweet wafer, stuffed biscuit or packaged cookie; 8) chocolate, ice cream, gelatin, flan or other industrialized desserts; 9) sausage, pork sausage, bologna or ham; 10) loaf of bread, hot dog or hamburger; 11) mayonnaise, ketchup or mustard; 12) margarine; 13) instant noodles, packet soup, frozen lasagna or another frozen ready-to-eat dish. Minimally and ultra-processed foods were counted continuously (e.g., the score of foods consumed per day).

### Presence of other comorbidities

The presence of diabetes was determined by the question “Has a doctor ever told you that you have diabetes?” (p. 130 of [20]) and the answer was given as binary variables ('yes' or 'no').

### Exercise and physical activity

Exercise and physical activity levels were determined from VIGITEL’s questionnaire, which includes questions related to physical activities performed in the domains of leisure (used to determine physical exercise levels), occupational, and transportation (p. 123–125 of [20]).

For the construction of indicators related to occupational and transport physical activity, the following questions were considered: 1) “Do you travel on foot or by bicycle to go to or return to work?”; 2) “How much time do you spend going back and forth on this route (on foot or by bicycle)?”; 3) “Currently, are you attending a course/school or taking someone to a course/school?”; 4) “To go or return to this course/school, do you walk or cycle?”; 5) “How much time do you spend going back and forth on this route (on foot or by bicycle)?”. 6) “In the last three months, have you worked?”; 7) “In your work, do you carry weight or do other intense activities?”; 8) “In a normal week, how many days do you do these activities at work?”; 9) “When you perform these activities, how long does it usually last?”. Exercise levels classification was considered by physical/sport activities performed in the domain of leisure (e.g., gait and fitness activities), with the question “In the last three months, did you engage in any type of physical exercise or sport?” [20].

Participants were classified as “non-practitioners = 0” or “practitioners = 1” regarding their participation in physical exercise/sport [14]. Subsequently, the sample was classified according to habitual physical activity (with the recommendations of the World Health Organization) [14]. For this purpose, all physical activities performed in the domains of leisure, occupational, and transportation [20] were considered and the participants were classified as “sufficiently active” or “insufficiently active” if the sum of weekly physical activity in these three domains was above or below 150 min/week, respectively [30].

### Statistical analysis

Descriptive statistical analysis considered measures of central tendency (mean, and 95% confidence interval [95% CI] of the mean) and relative frequency. Possible outliers were identified with the interquartile range (1.5) (considering BMI, education level, leisure exercise, and occupational and transportation physical activity [before categorizing as insufficiently or sufficiently active]). Binary logistic regression was used to verify the odds of older hypertensive adults being (or not) an exercise practitioner and sufficiently/insufficiently active. The classification of exercise (0 = non-practitioner; 1 = practitioner) and physical activity (0 = insufficiently active; 1 = sufficiently active) levels were considered as the dependent variables while sociodemographic characteristics, BMI, health risk behaviors, eating habits, and diabetes as independent variables. Four models were generated (crude: BMI; Model 1: BMI + sociodemographic characteristics; Model 2: BMI + sociodemographic characteristics + health risk behaviors; and Model 3: BMI + sociodemographic characteristics + health risk behaviors + eating habits + the presence of diabetes). The interpretation of the models was reported through odds ratio (OR). All analyses were performed using the SPSS, v. 20.0 (Inc., Chicago, IL, EUA), with a significance level previously established (α = 5%). This manuscript has been produced with the requirements of the Strengthening the Reporting of Observational Studies in Epidemiology (STROBE) checklist for cross-sectional studies.

## Results

**Fig. 1:**
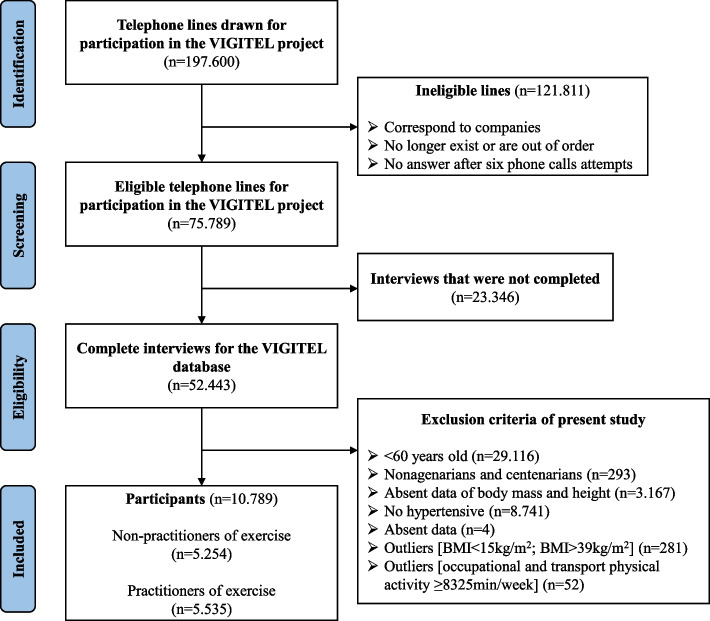
Flowchart of selection of subjects for this study. Abbreviations: VIGITEL = Surveillance of Risk Factors and Protection for Chronic Diseases by Telephone Survey; BMI = Body mass index

11.122 participants were eligible for the study. After the detection of outliers, 10.789 older adults were included in the final sample (Fig. 1). Of this sample, 48.7% (n: 5.254) were classified as exercise non-practitioner and 51.3% (n: 5.535) as exercise practitioners (Fig. 1; Table 1). Among exercise practitioners, 39.6% (n: 2.122) were classified as insufficiently active while 7.7% (n: 404) were sufficiently active among non-practitioners.

**Table 1:** Descriptive analysis of older hypertensive adults' exercise non-practitioners (*n* = 5.254) and practitioners (*n* = 5,535) and insufficiently (*n* = 6.977) and sufficiently active (*n* = 3.817), who participated in the project on surveillance of risk and protection factors for chronic diseases by telephone survey (VIGITEL)

**Variables**	**Total (*n* = 10.789)**			**Exercise non-practitioners(*n* = 5.254)**						**Exercise practitioners(*n* = 5.535)**					
				**Insufficiently active (*n* = 4.850)**			**Sufficiently active (*n* = 404)**			**Insufficiently active (*n* = 2.122)**			**Sufficiently active (*n* = 3.413)**		
	**Mean**	**95%CI**		**Mean**	**95% CI**		**Mean**	**95% CI**		**Mean**	**95% CI**		**Mean**	**95% CI**	
		**LL**	**UL**		**LL**	**UL**		**LL**	**UL**		**LL**	**UL**		**LL**	**UL**
**Sociodemographic**															
Age (years)	70.9	70.8	71.0	72.0	71.8	72.2	66.8	66.2	67.3	71.3	71.0	71.7	69.6	69.3	69.8
Years of study (years)	9.4	9.3	9.5	8.4	8.2	8.5	8.4	7.9	8.8	10.0	9.8	10.2	10.6	10.4	10.8
**Sex**															
Male (%)	31.7			26.9			45.6			27.9			39.4		
Female (%)	68.3			73.1			54.4			72.1			60.6		
**Anthropometry**															
Body mass (kg)	70.8	70.6	71.1	70.4	70.0	70.8	72.6	71.4	73.8	70.6	70.0	71.1	71.3	70.8	71.7
Height (m)	1.61	1.61	1.61	1.60	1.60	1.61	1.63	1.62	1.63	1.61	1.6	1.61	1.62	1.62	1.63
BMI (kg/m^2^)	27.2	27.1	27.3	27.4	27.2	27.5	27.4	27.1	27.8	27.2	27.1	27.4	27.0	26.8	27.1
**Physical activity**															
Occupational and transport (min/week)	55.7	50.1	61.4	2.7	2.2	3.2	814.0	724.2	903.8	0.5	0.2	0.8	75.6	64.6	86.7
Leisure (min/week)	93.4	91.1	95.8							72.2	70.6	73.7	250.5	247.2	253.8
Total	149.2	143.1	149.2	2.7	2.2	3.2	814.0	724.2	903.8	72.7	71.1	74.2	326.2	315.1	337.2
**Health risk behavior**															
Screen time (≥ 3 h/day) (%)	50.2			49.9			42.1			49.3			50.9		
Alcohol drinker (%)	75.2			80.9			69.1			75.5			67.5		
Smoker (%)	5.0			6.4			9.3			3.5			3.4		
**Eating habits**															
Minimally processed food consumption (score/day)	7.2	7.2	7.2	6.8	6.7	6.8	6.9	6.7	7.1	74.0	7.3	7.5	7.7	7.6	7.7
Ultra-processed food consumption (score/day)	1.9	1.8	1.9	2.0	1.9	2.0	2.1	1.9	2.2	1.9	1.8	1.9	1.7	1.7	1.8
**Presence of comorbidities**															
Diabetes (%)	26.3			28.4			22.6			28.9			23.9		

*CI* Confidence Interval, *LL* Lower limit, *UL* Upper limit, *BMI* Body mass index

Table 1 provides the total sample description according to exercise and physical activity levels regarding sociodemographic and anthropometric variables, physical activity (leisure, occupational, and transportation), health risk behaviors, eating habits, and the presence of diabetes. From the total sample, 49.2% are in the category 'overweight' (n: 5.308), and no significant differences were observed (*p* > 0.05) in BMI in the comparison between the groups of exercise non-practitioners vs. practitioners (avoiding the analysis bias in regression models). The frequency (percentage of older adults) related to the type of exercise performed by the participants in the practitioner group is shown in Supplementary Table 1.

Table 2 presents the logistic regression model, with OR of older hypertensive adults to be exercise practitioner and sufficiently active, in accordance with nutritional status (BMI), sociodemographic characteristics, health risk habits, eating habits, and the presence of diabetes.

**Table 2:** Binary logistic regression models with the association of nutritional status (BMI) and other factors on exercise participation (be or not a practitioner) and recommended amount of physical activity (sufficiently active)

Factors	*Models*							
	**Crude**		**1**		**2**		**3**	
	**OR**	**95% CI**	**OR**	**95% CI**	**OR**	**95% CI**	**OR**	**95% CI**
***Exercise (odds of be practitioner)***								
BMI (kg/m^2^)	0.984^*^	[0.975 – 0.993]	0.980^*^	[0.971 – 0.989]	0.976^*^	[0.967 – 0.985]	0.981^*^	[0.972 – 0.991]
**Sociodemographic characteristics**								
Age (years)			0.982^*^	[0.977 – 0.988]	0.981^*^	[0.976 – 0.986]	0.980^*^	[0.975 – 0.986]
Years of study (years)			1.068^*^	[1.060 – 1.075]	1.064^*^	[1.056 – 1.073]	1.050^*^	[1.041 – 1.058]
Sex (female: 0; male: 1)			1.297^*^	[1.194 – 1.410]	1.205^*^	[1.104 – 1.314]	1.247^*^	[1.140 – 1.363]
**Health risk habits**								
Screen time (≥ 3 h/day)					0.923^#^	[0.852 – 0.999]	0.947	[0.872 – 1.027]
Alcohol consumption (no: 0; yes:1)					0.717^*^	[0.651 – 0.790]	0.702^*^	[0.637 – 0.779]
Tobacco consumption (no: 0; yes:1)					0.421^*^	[0.349 – 0.507]	0.464^*^	[0.384 – 0.562]
**Eating habits**								
Minimally processed food consumption (score/day)							1.167^*^	[1.145 – 1.191]
Ultra-processed food consumption (score/day)							0.886^*^	[0.863 – 0.909]
**Presence of other comorbidities**								
Diabetes (no: 0; yes: 1)							0.831^*^	[0.759 – 0.909]
***Physical activity (odds of be sufficiently active ***^***†***^***)***								
BMI (kg/m^2^)	0.984*	[0.975 – 0.993]	0.973^*^	[0.963 – 0.982]	0.970^*^	[0.961 – 0.980]	0.975^*^	[0.965 – 0.985]
**Sociodemographic characteristics**								
Age (years)			0.956^*^	[0.950 – 0.962]	0.955^*^	[0.950 – 0.961]	0.955^*^	[0.949 – 0.961]
Years of study (years)			1.041^*^	[1.033 – 1.049]	1.038^*^	[1.030 – 1.046]	1.026^*^	[1.018 – 1.035]
Sex (female: 0; male: 1)			1.728^*^	[1.588 – 1.881]	1.580^*^	[1.446 – 1.728]	1.633^*^	[1.491 – 1.789]
**Health risk habits**								
Screen time (≥ 3 h/day)					0.982^#^	[0.811 – 0.959]	0.909^#^	[0.835 – 0.990]
Alcohol consumption (no: 0; yes:1)					0.697^*^	[0.632 – 0.769]	0.683^*^	[0.621 – 0.758]
Tobacco consumption (no: 0; yes:1)					0.557^*^	[0.457 – 0.679]	0.601^*^	[0.492 – 0.736]
**Eating habits**								
Minimally processed food consumption (score/day)							1.132^*^	[1.109 – 1.155]
Ultra-processed food consumption (score/day)							0.896^*^	[0.871 – 0.921]
**Presence of other comorbidities**								
Diabetes (no: 0; yes: 1)							0.780^*^	[0.708 – 0.859]

Model 1 was adjusted for sociodemographic variables; Model 2 was adjusted for sociodemographic variables and health risk behaviors; Model 3 was adjusted for sociodemographic variables, health risk behaviors, eating habits, and the presence of diabetes. †Older subjects are classified as having physical activity level ≥ 150 min/week of occupational and transport activities, or > 75 min of leisure activities, or a combination of leisure, occupational, and transport activities greater than 150 min/week

*OR* Odds ratio, *CI* Confidence interval, *BMI* Body mass index

^*^ *p* < 0.001

^#^ *p* < 0.05

All factors were associated with the OR of older hypertensive adults being exercise practitioners, except for screen time (Table 2). For each higher classification of BMI (OR: 0.981[95% CI: 0.972 – 0.991), age (OR: 0.980 [95% CI: 0.975—0.986]), alcohol (OR: 0.702 [95% CI: 0.637 – 0.779]) and tobacco consumption (OR: 0.464[95% CI: 0.384 – 0.562]) ultra-processed food consumption score (OR: 0.886[95% CI: 0.863 – 0.909]), and presence of diabetes (OR: 0.831[95% CI: 0.759 – 0.909]), there is a decrease of OR to be exercise practitioner (*p* < 0.001). The factors of male sex (OR: 1.247[95% CI: 1.140 – 1.363]), each year of schooling (OR: 1.050[95% CI: 1.041 – 1.058]) and minimally processed food consumption score (OR: 1.167 [95% CI: 1.145 – 1.191]) increase the OR to be exercise practitioner (*p* < 0.001).

All factors were associated with the OR of older hypertensive adults being sufficiently physically active. For each higher classification of BMI (OR: 0.975 [95% CI: 0.965 – 0.985]), screen time (OR: 0.909[95% CI: 0.835 – 0.990]), alcohol (OR: 0.683[95% CI: 0.621 – 0.758) and tobacco (OR: 0.601 [95% CI: 0.492 – 0.736]) consumption ultra-processed food consumption score (OR: 0.896[95% CI: 0.871 – 0.921]), and presence of diabetes (OR: 0.780[95% CI: 0.708 – 0.859]), there is a decrease of OR on older adults to be sufficiently active (*p* < 0.05). The factors of male sex (OR: 1.633[95% CI: 1.491 – 1.789), each year of schooling (OR: 1.026[95% CI: 1.018 – 1.035]) and minimally processed food consumption score (OR: 1.132 [95% CI: 1.109 – 1.155]) increase the OR to be sufficiently active, respectively (*p* < 0.001).

## Discussion

This is the first study that aimed to investigate the relationship between physical activity and exercise participation in older Brazilian hypertensive adults with nutritional status (BMI), health risk behaviors, eating habits, and the presence of diabetes. The results showed that BMI, age, screen time (only for physical activity), alcohol and tobacco consumption, ultra-processed food consumption scores, and having diabetes decreased the odds of being exercise practitioners or sufficiently active. On the other hand, male sex, years of study, and minimally processed foods increased the odds of older hypertensive adults being exercise practitioners or sufficiently active. Therefore, this study advances the investigation of hypertension and behavioral factors that are associated with exercise and physical activity participation of older adults in a region so far not investigated (Brazil).

Previous findings indicated that higher nutritional status (overweight/obesity) is associated with lower odds of compliance with exercise and physical activity [21, 22, 31]. In this sense, a higher nutritional status over the ideal body weight may be associated with lower odds of adherence to exercise programs and reduced habitual physical activity [32]. Consequently, deleterious behaviors tend to combine and co-exist (for example, higher sedentary behavior [increased exposure to cell phone, television, and computer; time sitting or lying] [33], alcohol [21], and tobacco consumption [31]). Moreover, inadequate eating habits (with high consumption of ultra-processed foods) are inversely related to sufficient physical activity levels (high consumption of ultra-processed foods and low physical activity levels) [34]. Recent findings reported that insufficiently active subjects consume 6% more ultra-processed foods (in calories) compared to sufficiently active subjects [34]. One possible explanation for this is that subjects with sufficient physical activity had more satiety with lower amounts of high-density food intake (e.g., ice cream, instant noodles) when compared with insufficiently active subjects, who needed a higher intake amount (of ultra-processed foods) to have satiety [35]. The need to ingest larger amounts of ultra-processed foods to satisfy satiety in insufficiently active subjects increases their odds of going over the daily caloric balance, favoring overweight and obesity [36]. Therefore, the interaction between nutritional status and associated risk factors (screen time, use of licit drugs, and inadequate eating habits) compromises the odds of older hypertensive adults being practitioners of exercise and complying with the recommendations of physical activity in daily life [37].

Another relevant finding from our study is the distribution of combinations of different exercise (non-practitioners/practitioners) and physical activity (insufficiently active/sufficiently active) characteristics of older hypertensive adults. Some older hypertensive adults presented sufficient weekly physical activity but did not engage in exercise (*n* = 404). Even older non-practitioners of exercise can be sufficiently active through occupational and transportation activities. Leisure activities (i.e., exercise) are not the only way in which older people can be sufficiently active. Therefore, these sufficiently active older adults may benefit from a more active lifestyle compared to insufficiently active older adults, as regular physical activity reduces the risk of coronary heart disease, obesity, and some cancers [6, 22]. This makes it valuable to develop simple strategies to increase all physical activity that can be performed whenever possible, such as using the stairs instead of the elevator, parking the car farther away, or getting off the bus before the final stop to walk to the destination [30].

On the other hand, this study demonstrated that some older hypertensive adults who engaged in exercise programs were classified as insufficiently active (39.6%). Although exercise is well recommended as an effective non-pharmacological mean of controlling blood pressure and decreasing cardiovascular risk, it is necessary to have an adequate weekly volume and frequency (Supplementary Chart 1) [37, 38]. The prescription of types of exercise (aerobic, strength, or combined) and variables (intensity, volume, frequency, and duration) must be assertively chosen by exercise professionals to maximize the benefits of each intervention [7, 9, 10, 37]. Therefore, in addition to raising awareness of the regular performance of exercise, the exercise prescription must be adjusted in volume and weekly frequency to maintain good health status for older hypertensive adults [39].

One strength of this study is the large number of participants, with a good sample representation from all Brazilian regions. Data collection through telephone interviews carried out by a highly qualified team allowed clear and precise explanations on each issue, avoiding misunderstandings. The identification of outliers minimized the analyses biased results (a discrepant value that could interfere with the confidence level of the results), homogenizing the sample [40, 41]. Somehow, outliers can under/overestimate the results of the analysis. For example, we analyzed the data without removing the outliers and the results provided were not similar to the current results. When the outliers were not removed, the OR of alcohol consumption was > 1 to be an exercise practitioner or sufficiently active in physical activity (Supplementary Table 2). In other words, alcohol consumption was positively associated with physical activity and exercise. However, when the outliers were removed, the OR was < 1 (Table 2). Previous literature [42, 43] demonstrates that alcohol consumption has a negative influence on older adults being exercise practitioners or sufficiently active. Therefore, our caution in considering discrepant values was carried out.

Another strength is the regression model adjustment with a wide range of confounding factors that influence the odds of exercise and sufficient physical activity minimized analyses’ bias. As a result, this study is the first to suggest that nutritional status, health risk behaviors, eating habits, and the presence of diabetes have an association with the odds of older hypertensive adults being exercise practitioners and sufficiently active in a developing country [22]. The authors considered that adequate physical activity was independently associated with sex (male), age, self-independence, lower psychological distress, rural residence, not having diabetes, adequate minimally processed food intake, and speaking a language other than English at home [22]. In contrast, our study discusses barriers to physical activity and exercise practice, and our findings suggest that some behaviors are associated with sufficient physical activity and exercise practice. Considering the previous findings, and the lack of literature about the associated factors to sufficient physical activity and exercise practice of older hypertensive adults, our results are promising.

This study also had limitations. VIGITEL is a telephone survey, which may be influenced by its methodological effect Some examples of methodological effect include the sampling type (Brazilian), questions (under/overestimation of questions [e.g., physical activity level]), means of data collection (telephone [not smartphone] line), interview time (longer-interviews tend to have more errors in some questions), and duration of the survey (reduced time to collect more data). Despite being comprehensive and representative, the cross-sectional design did not allow the establishment of cause-effect relationships. The extrapolation of these findings to populations from other countries should be considered with caution, considering other differences that can impact the odds of older adults being exercise practitioners and sufficiently active. The lack of cognitive assessment before the interview is a limitation because some answers could be misunderstood by the participants. The use of BMI to determine nutritional status also limited the study due to BMI not distinguishing body composition components. However, it is the most used epidemiological index in the world, being adequate and feasible for representative samples [44]. Another limitation is the lack of information on the presence of antihypertensive drugs and blood pressure values (for adjustment of logistic regression models). This information could, in a way, change the association of the older adult being an exercise practitioner and sufficiently active, based on the use of medication (yes/no, or quantity) and blood pressure measurement (given continuously, e.g., 120 × 80 mmHg). Another limitation is the no determination of association between the amount of tobacco and alcohol consumption on physical activity and exercise, in terms of daily amount and exposure time in years. Moreover, our study considered these variables in a categorical way (i.e., tobacco, alcohol, and screen time), preventing the test of multicollinearity and interaction between themselves. However, a study with other population (adolescents) showed positive associations between these variables (tobacco, alcohol, and screen time) [45]. The 24-recall method applied in only one day has a poorer association with total energy intake (*r* = 0.31) measured by the doubly labeled water (gold standard method) compared to the same method applied more times (two or more days; r between 0.39 and 0.47) [28]. Future studies need to consider more days of application of the 24-recall method. Finally, the physical activity assessment was carried out using a questionnaire from the VIGITEL study, which could under/overestimate the real level of physical activity performed by older adults. However, this questionnaire is validated against other common questionnaires (e.g., IPAQ; BAECKE) for physical activity assessment [46].

As practical implications of this study’s findings, health professionals responsible for the treatment and control of hypertension in older adults can educate older adults about the associated factors of exercise and physical activity participation. The magnitude of positive and negative associations of each factor (nutritional status, health risk behaviors, eating habits, and the presence of diabetes) may help change the lifestyle of older hypertensive adults. Future research should explore the underlying reasons for the frequency of health risk habits in insufficiently active older hypertensive adults to help the development of public health interventions. In the public health field, helping older hypertensive adults reach the physical activity recommendations will contribute to the reduction of healthcare services expenses [47]. One possibility for health professionals to stimulate older adults is to install and use applications for smartphones that measure and encourage physical activity (e.g., steps daily count). Recent evidence suggests that these devices may be effective in increasing physical activity in older adults (although larger trials with longer follow-ups are needed to clarify if these devices provide clinically important effects) [48, 49]. If massive intervention would be practiced, screen time could have a positive OR to be sufficiently active in the future. Moreover, health professionals’ knowledge may help with adequate exercise prescription, even if this prescription is done gradually.

From a multifactorial approach, many reasons could be associated with a reduced chance of being an exercise practitioner. Beyond what is explored in our study, other factors including accessibility to exercise (adequate space to exercise practice), monthly income (could lead to access to adequate spaces for exercise, even personalized service), and public policies promoted by the government [50] could be discussed. However, our study did not investigate these factors, and we cannot generalize the adoption of means of physical activity and exercise for all contexts. Therefore, more studies are needed to clarify this multifaceted phenomenon.

Additionally, we suggest the longitudinal analysis (cohort or intervention studies) of the associated factors and levels of exercise and physical activity of older hypertensive older adults to establish the causal relationships between the factors analyzed in our study and recommended amounts of physical activity and exercise. We also suggest reproducing the study in other countries for greater external validity, and as a result, generalization across other populations.

## Conclusion

Nutritional status, sociodemographic characteristics, health risk behaviors, eating habits, and the presence of diabetes are associated with recommended amounts of physical activity and exercise in older hypertensive adults. Higher BMI, age, screen time (only for physical activity), and alcohol, tobacco, ultra-processed food consumption score, and having diabetes are negatively associated with exercise and physical activity suitability in older hypertensive adults. Being a male, level of education and minimally processed food consumption score are positively associated with exercise and sufficient levels of physical activity. The findings from this study may help health professionals understand how these factors are associated with the increase/decrease of the odds of older hypertensive adults participating in exercise and being sufficiently active. In this sense, best strategies can be elicited for treatment and arterial hypertension control.

## Supplementary Information

**Additional file 1:** **Supplementary Table 1.** Percentage values (%) of exercise types practiced by hypertensive older adults’ from VIGITEL project (*n*=5.535).

**Additional file 2:** **Supplementary Table 2.** Binary logistic regression model (including outliers [*n*=333]; total sample=11,122) with the association of nutritional status (BMI) and other factors on exercise participation (be or not a practitioner) and recommended amount of physical activity (sufficiently active).

**Additional file 3:** **Supplementary Chart 1.** Summary of main benefits of exercise and physical activity and recommendations of corresponding intensity, duration, and frequency for older hypertensive adults.

## Data Availability

All data are public and can be found on the official website of the study https://www.gov.br/saude/pt-br/assuntos/saude-de-a-a-z/v/vigitel-1/vigitel.
