# Supplementary material for: Nutritional status, health risk behaviors, and eating habits are correlated with physical activity and exercise of brazilian older hypertensive adults: a cross-sectional study
Source: BMC Public Health. 2022 Dec 19;22:2382. doi: 10.1186/s12889-022-14873-4 (PMC9762644; doi:10.1186/s12889-022-14873-4)
Supplement: Supplementary file 1 — Additional file 1: Supplementary Table 1. Percentage values (%) of exercise types practiced by hypertensive older adults’ from VIGITEL project (n=5.535). [file 12889_2022_14873_MOESM1_ESM.docx]

**Supplementary Table 1.** Percentage values (%) of exercise types practiced by hypertensive older adults’ from VIGITEL project (n=5.535).

| **Physical exercise practiced** | **% of older adults practicians** |
| --- | --- |
| Walking | 53.0 |
| Walking in treadmill | 3.2 |
| Gym | 5.5 |
| Hydro gymnastic | 10.5 |
| General gymnastic | 13.7 |
| Swimming | 1.3 |
| Martial arts and fighting | 0.27 |
| Cycling | 3.7 |
| volleyball/footvolley | 0.14 |
| dance | 1.7 |
| Running | 1.0 |
| Running in treadmill | 0.33 |
| Aerobic gymnastic | 1.1 |
| Soccer/futsal | 0.83 |
| Basketball | 0.02 |
| Tennis | 0.14 |
| Other type of physical exercise | 3.4 |
