# Supplementary material for: Nutritional status, health risk behaviors, and eating habits are correlated with physical activity and exercise of brazilian older hypertensive adults: a cross-sectional study
Source: BMC Public Health. 2022 Dec 19;22:2382. doi: 10.1186/s12889-022-14873-4 (PMC9762644; doi:10.1186/s12889-022-14873-4)
Supplement: Supplementary file 2 — Additional file 2: Supplementary Table 2. Binary logistic regression model (including outliers [n=333]; total sample=11,122) with the association of nutritional status (BMI) and other factors on exercise participation (be or not a practitioner) and recommended amount of physical activity (sufficiently active). [file 12889_2022_14873_MOESM2_ESM.docx]

Supplementary Table 2. Binary logistic regression model (including outliers [n=333]; total sample=11,122) with the association of nutritional status (BMI) and other factors on exercise participation (be or not a practitioner) and recommended amount of physical activity (sufficiently active).

| **Factors** | ***Models*** | | | | | | | |
| --- | --- | --- | --- | --- | --- | --- | --- | --- |
|  | **Crude** | | **1** | | **2** | | **3** | |
|  | **OR** | **95% CI** | **OR** | **95% CI** | **OR** | **95% CI** | **OR** | **95% CI** |
| ***Exercise (odds of be practitioner)*** | | | | | | | | |
| BMI (kg/m^2^) | 0.979^*^ | [0.972 – 0.986] | 0.977^*^ | [0.970 – 0.985] | 0.975^*^ | [0.967 – 0.982] | 0.978^*^ | [0.970 – 0.986] |
| **Sociodemographic characteristics** |  |  |  |  |  |  |  |  |
| Age (years) |  |  | 0.982^*^ | [0.977 – 0.988] | 0.981^*^ | [0.976 – 0.986] | 0.981^*^ | [0.976 – 0.986] |
| Years of study (years) |  |  | 1.067^*^ | [1.059 – 1.074] | 1.063^*^ | [1.055 – 1.071] | 1.050^*^ | [1.042 – 1.059] |
| Sex (female: 0; male: 1) |  |  | 1.307^*^ | [1.204 – 1.419] | 1.211^*^ | [1.111 – 1.320] | 1.249^*^ | [1.144 – 1.363] |
| **Health risk habits** |  |  |  |  |  |  |  |  |
| Screen time (≥3h/day) |  |  |  |  | 0.916^#^ | [0.847 – 0.992] | 0.936 | [0.864 – 1.014] |
| Alcohol consumption (no: 0; yes:1) |  |  |  |  | 1.405^*^ | [1.276 – 1.546] | 1.428^*^ | [1.295 – 1.574] |
| Tobacco consumption (no: 0; yes:1) |  |  |  |  | 0.424^*^ | [0.352 – 0.510] | 0.472^*^ | [0.391 – 0.569] |
| **Eating habits** |  |  |  |  |  |  |  |  |
| Minimally processed food consumption (score/day) |  |  |  |  |  |  | 1.171^*^ | [1.149 – 1.194] |
| Ultra-processed food consumption (score/day) |  |  |  |  |  |  | 0.884^*^ | [0.861 – 0.907] |
|  |  |  |  |  |  |  |  |  |
| ***Physical activity (odds of be sufficiently active ^†^)*** | | | | | | | | |
| BMI (kg/m^2^) | 0.979* | [0.971 – 0.981] | 0.972^*^ | [0.963 – 0.980] | 0.970^*^ | [0.961 – 0.978] | 0.972^*^ | [0.964 – 0.981] |
| **Sociodemographic characteristics** |  |  |  |  |  |  |  |  |
| Age (years) |  |  | 0.956^*^ | [0.950 – 0.961] | 0.955^*^ | [0.949 – 0.961] | 0.955^*^ | [0.949 – 0.960] |
| Years of study (years) |  |  | 1.041^*^ | [1.034 – 1.049] | 1.038^*^ | [1.030 – 1.046] | 1.027^*^ | [1.019 – 1.036] |
| Sex (female: 0; male: 1) |  |  | 1.741^*^ | [1.600 – 1.893] | 1.589^*^ | [1.454 – 1.735] | 1.639^*^ | [1.499 – 1.792] |
| **Health risk habits** |  |  |  |  |  |  |  |  |
| Screen time (≥3h/day) |  |  |  |  | 0.878^#^ | [0.808 – 0.954] | 0.894 | [0.822 – 0.972] |
| Alcohol consumption (no: 0; yes:1) |  |  |  |  | 1.445^*^ | [1.312 – 1.593] | 1.463^*^ | [1.326 – 1.613] |
| Tobacco consumption (no: 0; yes:1) |  |  |  |  | 0.557^*^ | [0.458 – 0.677] | 0.616^*^ | [0.505 – 0.750] |
| **Eating habits** |  |  |  |  |  |  |  |  |
| Minimally processed food consumption (score/day) |  |  |  |  |  |  | 1.132^*^ | [1.110 – 1.156] |
| Ultra-processed food consumption (score/day) |  |  |  |  |  |  | 0.896^*^ | [0.872 – 0.921] |

Note: OR=Odds ratio; CI=Confidence interval; BMI=Body mass index. *=p<0.001; #=p<0.05. Model 1 was adjusted for sociodemographic variables; Model 2 was adjusted for sociodemographic variables and health risk behaviors; Model 3 was adjusted for sociodemographic variables, health risk behaviors, and eating habits. †Older subjects are classified as having physical activity level ≥150min/week of occupational and transport activities, or >75min of leisure activities, or a combination of leisure, occupational, and transport activities greater than 150min/week.
