# Supplementary material for: Nutritional status, health risk behaviors, and eating habits are correlated with physical activity and exercise of brazilian older hypertensive adults: a cross-sectional study
Source: BMC Public Health. 2022 Dec 19;22:2382. doi: 10.1186/s12889-022-14873-4 (PMC9762644; doi:10.1186/s12889-022-14873-4)
Supplement: Supplementary file 3 — Additional file 3: Supplementary Chart 1. Summary of main benefits of exercise and physical activity and recommendations of corresponding intensity, duration, and frequency for older hypertensive adults. [file 12889_2022_14873_MOESM3_ESM.docx]

**Supplementary Chart 1.** Summary of main benefits of exercise and physical activity and recommendations of corresponding intensity, duration, and frequency for older hypertensive adults.

| **Authors** | **Benefits to cardiovascular system** | **Recommendations** |
| --- | --- | --- |
| *Aerobic exercise* | | |
| Mazini Filho et al., 2011 | ↓16.1% Triglycerides  ↓13,5% Fasting blood glucose  ↓12% Total Cholesterol  ↓16.6 LDL  ↑3.9 HDL | ►Intensity: 12 to 13 of RPE; Session duration: 60-70 minutes; Weekly frequency: 3; Program duration: 52 weeks. |
| Nogueira et al., 2012 | ↓12.4% Systolic blood pressure  ↓14.8% Diastolic blood pressure  ↓3.6% Body mass index  ↓6.6% Fasting blood glucose | ►Intensity: 70-80% of VO_2_ Max; Session duration: 60 minutes; Weekly frequency: 3; Program duration: 10 weeks. |
| *Strength exercise* | | |
| Dantas et al., 2016 | ↑12.8% Total antioxidant capacity  ↓29.3% Plasma nitrite  ↑1.6% Basal vascular conductance  ↓4.2% Mean blood pressure | ► Intensity: 9-15 RM; Sets: 1-3 per exercise; Rest between sets: 60-120 seconds; Weekly frequency: 2-3; Program duration: 10 weeks. |
| Abrahin et al., 2022 | ↓8.1% Systolic blood pressure  ↓9.6% Double product | ► Intensity: 6-10 RM; Sets: 2 per exercise; Rest between sets: 90-120 seconds; Weekly frequency: 3; Program duration: 12 weeks. |
| *Combined exercise (both aerobic and strength exercise)* | | |
| Nogueira et al., 2012 | ↓20% Arterial stiffness | ► Intensity: 60-75% of MHR; Session duration: 60 minutes; Weekly frequency: 3; Program duration: 12 weeks. |
| Gimenes et al., 2015 | ↑15.5% 6-minute walking test  ↓2.9% Waist circumference | ► Intensity: 50-70% of MHR; Session duration: 90 minutes; Weekly frequency: 3; Program duration: 16 weeks. |
| Cassiano et al., 2020 | ↓13.1% LDL  ↑7.9% HDL  ↓7.8% Total Cholesterol | ► Intensity: 55-85% of MHR; Session duration: 60 minutes; Weekly frequency: 2; Program duration: 16 weeks. |
| *Physical activity* | | |
| Silva et al., 2021 | ↓7.6% Systolic blood pressure  ↓3.4% Diastolic blood pressure  ↓1.2% Neck circumference  ↓0.8% Waist circumference | ►Type: Physical activity in domains of work, active transport, housework, and leisure; Intensity: moderate/vigorous; Duration: 10 minutes continuous for 5 days/week or 150 min/week duration. |

Note: ↑= increase; ↓= decrease; LDL= low density lipoprotein; HDL= high density lipoprotein; RPE = rating perceived exertion Borg (6-20 points); MHR = Maximal heart rate; RM = repetition maximum; VO_2_ Max = maximum oxygen consumption.
